# Learning multiple routes in homing pigeons

**DOI:** 10.1098/rsbl.2014.0119

**Published:** 2014-04

**Authors:** Andrea Flack, Tim Guilford, Dora Biro

**Affiliations:** Department of Zoology, University of Oxford, South Parks Road, Oxford OX13PS, UK

**Keywords:** pigeon, route learning, sequential learning, learning and memory

## Abstract

The aerial lifestyle of central-place foraging birds allows wide-ranging movements, raising fundamental questions about their remarkable navigation and memory systems. For example, we know that pigeons (*Columba livia*), long-standing models for avian navigation, rely on individually distinct routes when homing from familiar sites. But it remains unknown how they cope with the task of learning several routes in parallel. Here, we examined how learning multiple routes influences homing in pigeons. We subjected groups of pigeons to different training protocols, defined by the sequence in which they were repeatedly released from three different sites, either sequentially, in rotation or randomly. We observed that pigeons from all groups successfully developed and applied memories of the different release sites (RSs), irrespective of the training protocol, and that learning several routes in parallel did not impair their capacity to quickly improve their homing efficiency over multiple releases. Our data also indicated that they coped with increasing RS uncertainty by adjusting both their initial behaviour upon release and subsequent homing efficiency. The results of our study broaden our understanding of avian route following and open new possibilities for studying learning and memory in free-flying animals.

## 1. Introduction

The lives of many birds centre on a focal point in the environment, the colony or roosting site. Their ability to return home from different places is therefore of great adaptive significance. Homing abilities have been most extensively studied in the pigeon, *Columba livia*. Thus, it has been shown that pigeons flying over familiar terrain come to rely on stereotypical routes when homing repeatedly from the same site [1]. These routes can be well characterized by two related measures, homing efficiency and track variation [1]. Pigeons carry out daily foraging flights to locations several kilometres away from the colony [2]. However, it is not yet clear how efficiently they cope with the task of learning multiple homing routes. Owing to their extraordinary capacity for visual discrimination [3–5], pigeons have been used to study learning and memory in the laboratory. Now, their skills at faithfully following routes open up new avenues to explore in-flight learning and memory phenomena. While simultaneous storage of multiple targets and associated routes is likely to be the norm for pigeons [2], we do not know the extent to which the amount of spatial information that has to be handled to home reliably from different sites compromises the efficiency with which such information can be gathered and subsequently applied during homing. Here, we asked how homing efficiency is being influenced by the number of homing routes to be learned in parallel. To answer this question, we used GPS technology to track the homing flights of pigeons repeatedly released from different sites either sequentially, in rotation or randomly.

## 2. Material and methods

We used 30 adult homing pigeons bred at the Oxford University Field Station (51°46′58.34″ N, 1°19′02.40″ W). All experimental birds were less than two years old and had not participated in any previous experiment. During releases, all subjects carried GPS logging devices attached to their back by a small Velcro strip glued to clipped feathers. For every flight, geographical longitude and latitude were logged by the devices at 1 Hz (i-gotU GT-120 Phototrackers, Mobile Action Technology, Inc., Taiwan; approx. 15 g). We released pigeons from three different release sites (henceforth RSs; electronic supplementary material, figure S1): Stonesfield (R1; distance and direction to home: 10.4 km, 144°), Weston-on-the-Green (R2; 10.2 km, 212°) and Beckley (R3; 10.2 km, 264°).

**Figure 1.**
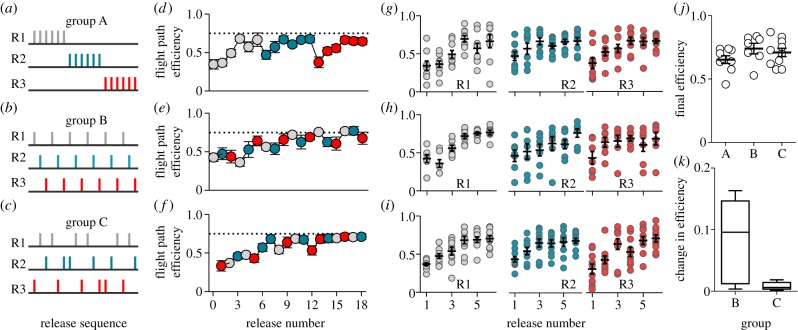
(*a*–*c*) Training protocols for the three experimental groups: A, B and C. (*d*–*f*) FPE (mean±s.e.m.) as a function of release for group A, B and C. Dashed line is a reference at 0.75. (*g*–*i*) FPE as a function of training at R1, R2 and R3 (*j*) FPE of the last three releases. Crosses show mean±s.e.m. (*k*) Change in FPE during the last five RS change (corresponds to last six releases) for B- and C-pigeons.

We used three different experimental groups of pigeons (A-, B- and C-pigeons). Each group experienced a distinct training protocol, defined by the sequence of releases from the three RSs (figure 1*a–c*). Training consisted of either (A) ‘sequential-site’ training (i.e. completing training at one site before commencing training at the next), (B) ‘rotation’ training (i.e. single releases cycling in a consistent order through R1, R2 and R3) or (C) ‘random-order’ training (i.e. releases alternating across the three RSs in a semi-random order). By the end of training, each bird had performed 18 homing flights (six per site). Training (maximum two releases per day) was conducted on consecutive days, interrupted only by unsuitable weather. One bird of group A and three birds of group B did not return to the loft.

All analyses were conducted in Matlab (Mathworks Inc.). We removed any point where the subject moved less than 0.5 m s^−1^, or any point after it reached within 250 m of the loft. Route acquisition was explored by analysing both flight path efficiency (FPE) and route fidelity (RF). FPE was the straight-line distance between the RS and the loft divided by actual travelled distance. RF was the mean nearest neighbour distance between all constituent points of two consecutive tracks. Also, we separately examined the pigeons' orientation behaviour at the RS (circling) and their overall homing behaviour (homing). Circling was the distance flown before leaving the surroundings of the RS (an area with a 2000 m radius centred at the RS), whereas homing was the distance flown in between leaving the RS's surroundings and reaching the loft.

## 3. Results

Route development during the first releases from a given spot in the field is characterized by increasing FPE and decreasing track variation (or increasing RF). We found a negative correlation between FPE and track variation (*p* = 0.001, *R* = −0.75, Pearson's). Still, as track variation is still relatively high after only six releases, we used FPE values in our comparisons. First, we asked how the different training protocols and RSs (figure 1*a*–*c*) influenced homing efficiency during training. We observed that, as training progressed, A-pigeons increased their FPE at all three sites (figure 1*d*), and that their FPE values from the 6th, 12th and 18th releases (the last ones at each site) did not differ across sites (repeated-measures-ANOVA, *p* = 0.94, *F*_2,16_ = 0.06). Also, in A-pigeons FPE increased equally at all three RSs (figure 1*g*). Interestingly, RS change did cause an average 25% (±4%) drop in FPE. Such a drop is thought to be caused by a mismatch between an expected constellation of external cues experienced by the birds upon release (which they learned through their previous experience at the site and use to determine their current position relative to that of the loft) and the actual constellation of cues found at the newly experienced field spot.

We therefore asked whether such a drop in homing efficiency would be compromised in a second group of pigeons (group B) trained to home in parallel (‘rotation’) from all three sites. We reasoned that, by compelling them to—gradually and sequentially—learn the constellations of external cues associated with each RS, the magnitude of the mismatch to be experienced upon any given RS change (and therefore also the drop in FPE) would be absent or significantly reduced. Also, we asked whether sequential releases from multiple RSs would slow down the increase in FPE typically observed during training, as the birds would be exposed to increased demands of handling navigational information. B-pigeons showed a similar FPE increase at all three sites (figure 1*h*). Furthermore, as training progressed, they reached similar FPE levels at approximately the same rate, when compared with A-pigeons (figure 1*d,e*). These results indicated that B-pigeons also learned the constellations of external cues associated with each site, and that they efficiently handled increased demands of navigational information. The question arose, therefore, whether it was the fixed sequence of releases that helped them reduce the mismatch between the expected and observed cues, or not.

To address this directly, we exposed a third group of pigeons (group C) to a series of random releases from all three sites, thereby preventing them from anticipating external cues to be experienced upon release. Intriguingly, C-pigeons reached the same maximum FPE levels observed in A- and B-pigeons (Kruskal–Wallis, *p* = 0.26, *K* = 2.71; figure 1*j*). Also, they exhibited more regular FPE levels during their last releases, when compared with B-pigeons. As a result, in spite of having been exposed to unpredictable RS changes, their FPE values showed an average variation of only 0.008 (±0.003) across consecutive releases, which was significantly lower than that of B-pigeons (*t*-test, *p* = 0.04, *t*_8_ = 2.40; figure 1*k*). From these observations, we concluded that C-pigeons also learned efficiently the constellations of cues associated with each of the three sites, even though they lacked the possibility to anticipate the location of the next release.

**Figure 2.**
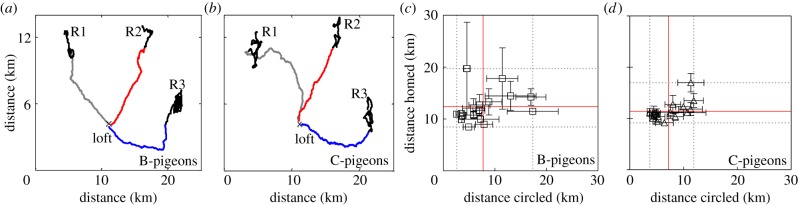
(*a*,*b*) Example tracks of B- and C-pigeons from R1, R2 an R3 in grey, red and blue, respectively. Black lines indicate circling at the RS. (*c*,*d*) Relationship between circling and homing distance for each release of B- and C-pigeons after the first RS change. Solid red and dotted grey lines indicate mean and ranges for each axis, respectively.

We then decided to further examine how B- and C-pigeons coped with continuous RS changes by comparing their circling values from both groups. Although both groups gave similar means (B-pigeons, 7719 m; C-pigeons, 7217 m), circling was more variable in B- than in C-pigeons (coefficient of variation: 58.5% and 40.1% in B- and C-pigeons, respectively). A similar pattern arose when we compared the homing distance between both groups. Both B- and C-pigeons had similar means (B-pigeons, 12384 m; C-pigeons, 11410 m), but the coefficient of variation of homing distance was larger in B- (24.3%) than in C-pigeons (15.9%). Lower and more regular circling values may be indicative of an active response to cope efficiently with increased navigational uncertainty, thereby reducing homing variations. We asked whether the lower variations of circling and homing in C-pigeons would help us detect a correlation between both measures. We found that C-pigeons showed a positive correlation between circling and homing distance (*p* = 0.006, *R*^2^ = 0.41, Pearson's, figure 2), whereas B-pigeons did not (*p* = 0.25). Taken together, these results showed that the same set of input conditions (i.e. RSs) led to different output phenomena in C-pigeons, namely, less variable and correlated circling and homing distance. In a behavioural context, such a phenomenon is thought to underlie reversible motivational changes triggered by external stimulation. Also, it is generally accepted that a state of higher motivation can lead to more efficient regulation of behavioural processes and less variable goal-directed actions [6]. We hypothesize that higher levels of uncertainty arising from unpredictable RS changes might have enhanced the motivation of C-pigeons to gather navigational information while orienting at the RSs, and use it subsequently to maximize homing efficiency.

## 4. Discussion

In nature, pigeons carry out daily foraging flights to up to 10 different locations [2]. They are known for their route following abilities, a phenomenon that involves following chains of landmarks, each of which triggers place recognition and recall of specific onward instructions [7]. In this study, pigeons were compelled to learn not only one, but three, sets of sequential cues. Furthermore, whereas A-pigeons experienced one homing route after the other, B- and C-pigeons had to learn all three routes simultaneously, either in a fixed or random order, respectively. By analysing high fidelity areas of trajectories, Mann *et al*. [8] identified habitual route waypoints, where multiple tracks of the same bird converged over salient visual features. They revealed that a bird requires only a small number of such waypoints to memorize its route. This proposition is supported by tests in the laboratory showing that pigeons can memorize a thousand pictorial stimuli [5] and recall the order of artificial stimuli [3,9]. It is therefore reasonable to assume that the boundaries of a pigeon's capacity to handle spatial memories lie beyond the challenge imposed by the three homing routes tested in this study. Moreover, using serial-probe-recognition tasks, pigeons have been used to study various learning phenomena, such as interference, primacy and recency effects [4,9]. Here, we examined their learning and memory capacities by training them to solve navigational tasks. Our data specified that, despite the different training protocols, all experimental groups successfully stored, recalled and applied route memories from all three sites. Importantly, B- and C-pigeons effectively applied route memories from past flights, even when they had recently homed from either one or two other sites. Also, our results showed that C-pigeons exhibited lower FPE variation than B-pigeons, despite the fact that they experienced the same number of RSCs. We suggest two possible explanations for this. First, uncertainty about the next RS may have increased the motivation of C-pigeons to detect and use landmarks more efficiently. Alternatively, the combination of a semi-randomized sequence of releases and consecutive releases from the same site (which occurred twice; figure 1*c*) might have strengthened RS memories in C-pigeons. Nonetheless, further experiments are required to test these hypotheses systematically.

Our results broaden our understanding of learning in birds by specifying how pigeons can learn and anticipate sets of sequential cues in flight. They open new possibilities for studying complex learning and memory phenomena in flying navigators.

## Data accessibility

Data available from the dryad digital repository: http://dx.doi.org/10.5061/dryad.j444n.
